# SARS-CoV2 RT-PCR assays: In vitro﻿ comparison of 4 WHO approved protocols on clinical specimens and its implications for real laboratory practice through variant emergence

**DOI:** 10.1186/s12985-022-01784-4

**Published:** 2022-03-28

**Authors:** Mariem Gdoura, Imen Abouda, Mehdi Mrad, Imen Ben Dhifallah, Zeineb Belaiba, Wasfi Fares, Anissa Chouikha, Maroua Khedhiri, Kaouther Layouni, Henda Touzi, Amel Sadraoui, Walid Hammemi, Zina Meddeb, Nahed Hogga, Sihem Ben Fadhel, Sondes Haddad-Boubaker, Henda Triki

**Affiliations:** 1 Faculty of Pharmacy of Monastir, University of Monastir, Monastir, Tunisia; 2 Laboratory of Clinical Virology, WHO Reference Laboratory for Poliomyelitis and Measles in the Eastern Mediterranean Region, Institut Pasteur de Tunis, University Tunis El Manar, Tunis, Tunisia; 3 Research Laboratory « Virus, Vectors and Hosts: One Health Approach and Technological Innovation for a Better Health» LR20IPT02, Institut Pasteur de Tunis, University Tunis El Manar, Tunis, Tunisia; 4 Laboratory of Biochemistry and Hormonology, Institut Pasteur de Tunis, University Tunis El Manar, Tunis, Tunisia; 5 Laboratory of Biomedical Genomics and Oncogenetics (LR16IPT05), Institut Pasteur de Tunis, University Tunis El Manar, Tunis, Tunisia

**Keywords:** SARS-CoV2, COVID-19, RT-PCR, Variant, Diagnosis, Sensitivity, In vitro, False positive, False negative

## Abstract

**Introduction:**

RT-PCR testing on nasopharyngeal swabs is a key component in the COVID-19 fighting, provided to use sensitive and specific SARS-CoV2 genome targets. In this study, we aimed to evaluate and to compare 4 widely used WHO approved RT-PCR protocols on real clinical specimens, to decrypt the reasons of the diverging results and to propose recommendations for the choice of the genome targets.

**Methods:**

260 nasopharyngeal samples were randomly selected among the samples tested between Week-16, 2020 and week-16 2021, in the Institut Pasteur de Tunis, Tunisia, one of the referent laboratories of COVID-19 in Tunisia. All samples were tested by Charité, Berlin protocol (singleplex envelop (E) and singleplex RNA-dependent RNA polymerase (RdRp)), Hong Kong Universiy, China protocol (singleplex nucleoprotein (N) and singleplex Open reading frame Orf1b), commercial test DAAN Gene® (using the CDC China protocol), (triplex N, Orf1ab with internal control) and Institut Pasteur Paris protocol (IPP) (triplex IP2(nsp9) and IP4 (nsp12) with internal control). For IPP, a selection from samples positive by IP2 but negative with IP4 was re-tested by exactly the same protocol but this time in singleplex. New results were described and analyzed.

**Results:**

In vitro analysis showed discordant results in 29.2% of cases (76 out of 260). The most discordant protocol is DAAN Gene® due to the false positive late signals with N target. Discordant results between the two protocol’s targets are more frequent when viral load are low (high Ct values). Our results demonstrated that the multiplexing has worsened the sensitivity of the IP4 target.

**Conclusion:**

We provide concise recommendations for the choice of the genome targets, the interpretation of the results and the alarm signals which makes suspect a gene mutation.

## Introduction

The Severe Acute Respiratory Syndrome Coronavirus 2 (SARS-CoV2) is responsible for the coronavirus disease (COVID-19). Rapidly after its emergence at the end of 2019 in Wuhan, China, COVID-19 has being spread globally and becoming a massive pandemic [1, 2]. COVID-19 pandemic has provided a great recognition to the key role played by the laboratories in the diagnostic testing; for screening, monitoring and contact-tracing [3]. Indeed, molecular testing is the angular stone to prevent and control virus circulation: it is reliable, rapid and accurate. Through this pandemic, millions of RT-PCR tests were carried out daily, which demonstrate how suitable it is for large scale testing. As of 29 September 2021, 244.69 per 1000 inhabitants were tested by RT-PCR in Tunisia (total of 2.92 millions) which is a high rate as compared to other countries [4]. Soon after the emergence of the SARS-CoV2, the first full sequence was published on January 10th, 2020 and the first RT-PCR detection assay was published on January 23rd, 2020 [5]. Today, whole panoply of protocols and kits is available within reach of all laboratories; many other protocols have been published by research groups, number of them has been approved by the WHO in March 2020 and a huge number of commercial tests have been created by companies [6, 7]. These tests are diverse by targeting a range of SARS-CoV2 specific genome regions. Obviously, genome targets impact the analytical performances; the sensibility of detection by RNA copy per milliliter and the specificity to detect unambiguously the SARS-CoV2 [3]. The biggest challenge for laboratories was to have a sufficiently sensitive RT-PCR assay to be able to detect the virus in pre-symptomatic individuals, who harbor often low viral loads [8]. Oppositely, high sensitivity may lead to false positive results especially for patients having recovered from SARS-CoV2 infection, due to genomic debris but no viable virus [9]. Many authors focused on the evaluation and the improvement of available RT-PCR protocols and tests, but especially for commercial tests.

In this study, we aimed to evaluate and to compare 4 widely used WHO approved RT-PCR protocols on real clinical specimen, to decrypt the origins behind the diverging results and to propose recommendations for the choice of genome targets. We focused on the impact of the SARS-CoV2 Alpha variant of cocern (VOC) detection by the Institut Pasteur Paris protocol and how to manage it.

## Methods

### Evaluation and comparison

The Laboratory of Virology of the Institut Pasteur de Tunis, Tunisia, is one of the national referent laboratories for the molecular and serological diagnosis of SARS-CoV2 since March 2020 and one of the national referent laboratories for the SARS-CoV2 variant emerging surveillance in Tunisia. From the bio-bank that contains remnant and retrospective clinical samples previously collected from individuals suspected to have, or diagnosed with SARS-CoV2 and stored at − 80 °C, we randomly selected 260 nasopharyngeal samples. These samples were collected between 13/04/2020 and 19/04/2021 (Week-16 2020 and week-16 2021), covering a year of virus circulation in Tunisia, through which, Alpha VOC  was introduced.

All samples underwent, first, RNA extraction using the QIAamp Viral RNA Mini Kit (QIAGEN®, Germany) then were tested, during 2 months period (May–June 2021)by the 4 WHO approved protocols compared in the present study [7]: Charité, Berlin protocol (singleplex envelop (E) and singleplex RNA-dependent RNA polymerase (RdRp), Hong Kong Universiy, China protocol (singleplex nucleoprotein (N) and singleplex open reading frame (Orf1b)), CDC China protocol (commercial test DAAN Gene ®, (triplex N, Orf1ab with internal control) and Institut Pasteur Paris protocol (triplex IP2 (nsp9) IP4 (nsp12) with internal control). (for the rest of the manuscript they will be called BERLIN, HKU, DAAN Gene®and IPP, respectively). Between different RT-PCR tests, the extracted RNAs were stored at + 4 °C for periods less than 24 h and at − 80 °C for longer periods. The interpretation of the results was done according to the respective protocol’s instructions. Interpretation took into consideration both the Cycle threshold (Ct) value and the shape of the obtained curves. Overall, when both targets per protocol gave positive amplification (Ct value ≤ 39), the result was positive, when both gave negative amplification (Ct value > 39), the result was negative, however, when just one target per 2 gave a positive amplification, the result was retained as "partial positive". Each protocol results were described and analyzed.

### Comparison between the protocols

We conducted a comparison between the 4 WHO approved protocols taking into consideration at least one positive target per protocol. Concordant and discordant results were rigorously classified and deeply analyzed. Then we compared the 8 targets independently of the protocols.

### Focus on IPP protocol

A selection from "partial positive" samples was retested by exactly the same protocol but this time in singleplex. New results were described and analyzed.

### Statistical analysis

The MedCalc® V18.2.1 Software was used for comparative analysis of the final results with the different RT-PCR protocols. The agreement between the different protocols' results was determined by the Cohen’s Kappa coefficient. The concordance between two observations increases when Kappa coefficient is closer to 1. The concordance between the targets' results was evaluated as well as the Ct values pairwise comparisons among the 4 protocols. The correlation between the Ct values of different targets was determined by the Spearman rank correlation coefficient. The calculation of the mean, median, standard deviation, maximum and minimum Ct values for each target was also performed. For the comparison of the two targets in each protocol, the t-test was used for the pairwise comparison of their Ct values. A *p*-value equal to or less than 0.05 was considered as statistically significant.

## Results

### Description of the results for each protocol

For the 260 samples, results for each protocol were represented in Table 1. The protocol that gave the most positive results was DAAN Gene® (55% positive results), the one that gave the most negative results was HKU (50.4% negative results) and the one that gave the most partial positive results was IPP (16.9%). Ct values for both targets of DAAN Gene®and BERLIN were different (*p* < 0.0001) the mean of difference was 1.5 ± 2 and 2.3 ± 2 respectively. The Ct value of BERLIN E was often lower than the BERLIN RdRP one and DAAN Gene®N was often lower than DAAN Gene®Orf 1ab. However, HKU and IPP gave similar Ct values for both targets (*p* > 0.001). For "partial positive" results, the targets that gave usually positive signals were E for BERLIN, N for HKU, N for DAAN and IP2 for IPP. In these partial positive results, the Ct values of the positive target were high for all the cases ( Ct value mean higher than 33.5) except for the IP2 (Ct value mean equal to 25.2).

**Table 1:** Description of the obtained RT-PCR results by protocol

	Protocols and targets	BERLIN	BERLIN E	BERLIN RdRp	HKU	HKU N	HKU Orf1b	DAAN Gene®	DAAN Gene® N	DAAN Gene® Orf1ab	IPP	IPP IP2	IPP IP4
“Positive”: target amplification by both targets per protocol	POSITIVE n(%)	n = 116 (44.6%)	116 (100%)	116 (100%)	n = 112 (43.1%)	112 (100%)	112 (100%)	n = 143 (55%)	143 (100%)	143 (100%)	n = 89 (35%)	89 (100%)	89 (100%)
	Ct value Median [min, max]		29 [16.39]	31 [18.39]		28[12.39]	27.5[16.39]		25[13.39]	28[16.39]		27[15.38]	27[15.39]
	Ct value mean +/− SD		28.9 +/− 5.2	30.5 +/− 4.8		27.4 +/− 5.9	27.5 +/− 5.9		26.2 +/− 6.6	28.5 +/− 6.1		27.4 +/− 5.8	27.4 +/− 6.4
	MEAN PAIRED DIFFERENCE +/− SD		1.5 +/− 2			0.2 +/− 1.9			2.3 +/− 2			0.04 +/− 1.6	
	PAIRED SAMPLE t-TEST		*p* < 0.0001			*p* = 0.3			*p* < 0.0001			*p* = 0.8	
« Partial positive» Target amplification by one target per protocol	POSITIVE n (%)	n = 17 (6.5%)	15 (88.2%)	2 (11.7%)	n = 17 (6.5%)	15 (88.2%)	2 (11.7%)	n = 39 (15%)	37 (94.9%)	2 (5.1%)	n = 44 (16.9%)	38 (86.4%)	6 (13.6%)
	Ct value Median [min, max]		37[24. 39]	33.5[33. 34]		36[31.38]	38[37.39]		38[35.39]	39[39.39]		24.5[15.38]	33.5[32.38]
	Ct value mean +/− SD		36 +/− 3.8	33.5 +/− 0.7		36 +/− 1.9	38 +/− 1.4		37.7 +/− 1.2	39 +/− 0		25.2 +/− 6.4	34.3 +/− 2.2
	NEGATIVE n (%)		2 (11.7%)	15 (88.2%)		2 (11.7%)	15 (88.2%)		2 (5.1%)	37 (94.9%)		6 (13.6%)	38 (86.4%)
« NEGATIVE» No target amplification by both targets per protocol, n(%)		n = 127 (48.8%)	127 (100%)	127 (100%)	n = 131. (50.4%)	131 (100%)	131 (100%)	n = 78 (30%)	78 (100%)	78 (100%)	n = 125 (48.10%)	127 (100%)	127 (100%)
Total		260 (100%)	260 (100%)	260 (100%)	260 (100%)	260 (100%)	260 (100%)	260 (100%)	260 (100%)	260 (100%)	260 (100%)	260 (100%)	260 (100%)

Charité, Berlin protocol (BERLIN): Berlin E and Berlin RdRp, University of Hong Kong protocol (HKU): HKU N and HKU Orf 1b, DAAN Gene® protocol (DAAN Gene®): DAAN Gene® N and DAAN Gene® Orf1ab, The Institut Pasteur, Paris (IPP) protocol: IPP IP2 and IPP IP4. Ct value: cycle threshold value, min: minimum, max: maximum, SD: standard deviation

### Evaluation of the concordance between the 4 protocols

#### Positive and negative concordance

Concordance and different groups of discordant results were represented in Table 2. All protocols gave the same results for 184 samples (70.8%): 72 concordant negative and 112 concordant positive. For positive samples, the obtained Ct values ranged from 12 to 39, (median = 28, mean = 27.5 ± 6.4).

**Table 2:** Description of the obtained results by group of samples: concordant positive, concordant negative and discordant groups

	DAAN Gene®	HKU	IPP	BERLIN	n
**Concordant: n = 184 (70.8%)**					
C1: Negative No amplification by all protocols	−	−	−	−	72
C2: Positive Amplification by all protocols (“positive” and “partial positive” results)	+	+	+	+	112
**Discordant: n = 76 (29.2%)**					
Group D1: Amplification by 3 protocols over 4 n = 21 (27.6%)	**+**	**+**	**+**	**−**	4
	**+**	**+**	**−**	**+**	6
	**+**	**−**	**+**	**+**	7
	**−**	**+**	**+**	**+**	4
Group D2: No amplification by 3 protocols over 4 n = 44 (57.9%)	**−**	**−**	**−**	**+**	0
	**−**	**−**	**+**	**−**	0
	**−**	**+**	**−**	**−**	1
	**+**	**−**	**−**	**−**	43
Group D3: Amplification by 2 protocols over 4 n = 11 (14.5%)	**+**	**+**	**−**	**−**	2
	**−**	**−**	**+**	**+**	1
	**+**	**−**	**+**	**−**	5
	**−**	**+**	**−**	**+**	0
	**+**	**−**	**−**	**+**	3
	**−**	**+**	**+**	**−**	0

Charité, Berlin protocol (BERLIN), University of Hong Kong protocol (HKU), DAAN Gene® protocol (DAAN Gene®), The Institut Pasteur, Paris (IPP) protocol

#### Discordance

The 76 remaining samples (29.2%) gave different discordant results being totally negative by at least one protocol and clearly positive by at least one protocol. The Ct values of positive targets for this group ranged from 13 to 39  (median = 36, mean = 33 ± 8.9) and were significantly higher than those of the concordant group (*p* < 0.0001). These 76 samples divided into 3 groups (Table 2): Group D1 contained 21 samples that gave positive results with 3 out of 4 protocols, almost equally distributed between the 4 protocols: HKU, IPP,  DAAN Gene® and BERLIN failed to detect 7, 6, 4 and 4 samples, respectively. Group D2 contained 44 samples that gave negative results with 3 out of 4 protocols and a positive result with only one protocol (DAAN gene in most of cases, 43 out of 44). Group D3 contained 11 samples that gave a positive result by two protocols and a negative result by two protocols.

To further focus on concordance between protocols, we calculated agreement between the results as represented in Table 3. The strength of agreement was very good and similar for the couples BERLIN/HKU, IPP/BERLIN and IPP/HKU, however, agreement was moderate between DAAN Gene®and the three other protocols.

**Table 3:** Evaluation of the agreement between the results by protocol using the Cohen’s Kappa agreement coefficient

	BERLIN	HKU	DAAN Gene®
IPP	**0.861** **95% CI [0.800–0.923]**	**0.830** **95% CI [0.763–0.898]**	0.542 95% CI [0.447–0.637]
DAAN Gene®	0.5419 95% CI [0.447–0.637]	0.5169 95% CI [0.422–0.611]	
HKU	**0.861** **95% CI [0.799–0.923]**		

Charité, Berlin protocol (BERLIN), University of Hong Kong protocol (HKU), DAAN Gene® protocol (DAAN Gene®), The Institut Pasteur, Paris (IPP) protocol. 95% CI: Confident interval of 95%, Concordance between two observations increases when Kappa coefficient is closer to 1.bold cases indicate good agreement

### Deep evaluations of the 8 targets independently of the protocol

**Fig. 1:**
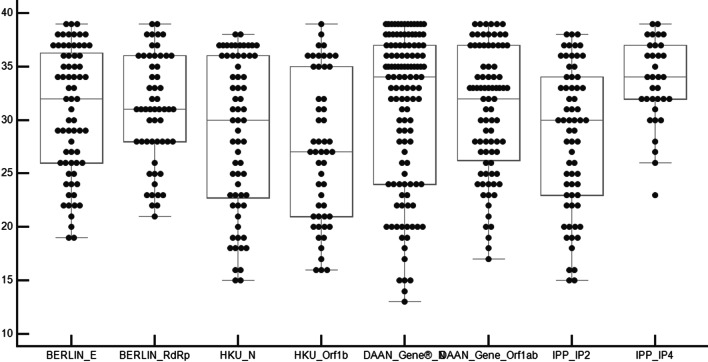
Ct values distribution per target. Box-and-Whisker plot: the central box represents the values from the lower to upper quartile (25 to 75 percentile). The middle line represents the median. A line extends from the minimum to the maximum value, excluding "outside" and "far out" values which are displayed as separate points

In order to evaluate the 8 targets regardless of their respective protocols, the Ct values for each target for the discordant results were represented in Fig. 1. The lowest Ct value was obtained by DAAN Gene®N target (Ct = 13) followed by the  IP2 target (Ct = 15). The lowest means were those of  IP2 (Ct = 27) followed by HKU N and HKU Orf 1b (Ct = 28). Generally, when the samples were amplified by the 8 targets, the samples with low viral load had high Ct values with all of them and those with high viral load gave low Ct values with all of them. The mean variation was of 7Ct ± 2.7 for each sample.

A big panel of discordant patterns was found between the 8 targets. The most interesting patterns were the divergent DAAN Gene®N and the IP4 from all others. The DAAN Gene®N target gave positive signal for 28 cases (Ct value median of 38 [36–39]) whereas the 7 other targets were negative. DAAN Gene®N target had a poor agreement with all other targets (Table 4). The IP4 gave negative signal for 30 cases whereas the 7 other targets gave relatively low Ct values (mean of 25.4 ± 5.3). IP4 had a poor agreement with all other targets (Table 4). For all targets combinations (except for those with IP4 or DAAN Gene®N or DAAN Gene®Orf 1ab) the agreement between qualitative results was almost good. However, the best agreements were noted for the combinations between a structural target and a non structural target: the original combinations [BERLIN E with BERLIN RdRp] and [HKU N with HKU Orf 1b] along with two new combinations [BERLIN E with IP2] and [BERLIN E with HKU Orf 1b]. For these new combinations, we performed pairwise comparison between the Ct values and found that, IP2 and HKU Orf 1b targets were more sensitive than BERLIN E as they gave lower Ct values than BERLIN E (*p* < 0.0001), the mean of difference being 3.3 ± 2.5 and 1.3 ± 2.5, respectively.

**Table 4:** Evaluation of the agreement between the results by protocol targets using the Cohen’s Kappa agreement coefficient

	Protocol	BERLIN		HKU		DAAN Gene®		IPP	
Protocol	Target	BERLIN E	BERLIN RdRp	HKU N	HKU Orf1b	DAAN Gene® N	DAAN Gene® Orf1ab	IPP IP2	IPP IP4
BERLIN	BERLIN E	**1**							
	BERLIN RdRp	**0.732** **95% CI [0.617–0.848]**	**1**						
HKU	HKU N	**0.713** **95% CI [0.591–0.836]**	**0.668** **95% CI [0.539–0.796]**	**1**					
	HKU Orf	0.476 95% CI [0.324–0.628]	0.547 95% CI [0.399–0.694]	**0.732** **95% CI [0.615–0.848]**	**1**				
DAAN Gene®	DAAN Gene® N	− 0.012 95% CI [− 0.104 to 0.080]	0.022 95% CI [− 0.054 to 0.098]	0.455 95% CI [0.305–0.604]	− 0.041 95% CI [− 0.119 to 0.037]	**1**			
	DAAN Gene® Orf1ab	0.476 95% CI [0.324–0.628]	0.463 95% CI [0.325–0.601]	0.455 95% CI [0.305–0.604]	0.353 95% CI [0.213–0.493]	0.156 95% CI [0.025–0.287]	**1**		
IPP	IPP IP2	**0.681** **95% CI [0.554–0.809]**	0.541 95% CI [0.396–0.686]	0.587 95% CI [0.445–0.728]	0.542 95% CI [0.399–0.685]	− 0.036 95% CI [− 0.123–0.051]	0.391 95% CI [0.236–0.545]	**1**	
	IPP IP4	0.240 95% CI [0.102–0.370]	0.011 95% CI [− 0.150 to 0.172]	0.124 95% CI [− 0.025 to 0.274]	− 0.056 95% CI [− 0.219 to 0.106]	− 0.006 95% CI [− 0.060 to 0.048]	0.117 95% CI [− 0.002 to 0.237]	0.312 95% CI [0.169–0.454]	**1**

Charité, Berlin protocol (BERLIN): Berlin E and Berlin RdRp, University of Hong Kong protocol (HKU): HKU N and HKU Orf 1b, DAAN Gene® protocol (DAAN Gene®): DAAN Gene® N and DAAN Gene® Orf1ab, The Institut Pasteur, Paris (IPP) protocol: IPP IP2 and IPP IP4. 95% CI: Confident interval of 95%, Concordance between two observations increases when Kappa coefficient is closer to 1.bold cases indicate good agreement

### Focus on IPP

The protocol  that gave the most partial positive results (44 samples out of 260, 16.4%) and for which Ct values of the the positive target is very low, is IPP. We randomly selected 26 samples that gave a positive high signal by IP2 and a negative signal by IP4. These 26 samples were re-tested by the same primers but this time, in singlelex. Results showed simplexing offers similar Ct values for IP2 but lower Ct values for the IP4 target, i.e. the sensitivity of IP4 was improved considerably (paired samples t-test is statistically significant (paired difference 10 ± 4.7 and p < 0.0001)).

## Discussion

In the present study, we provided a concise description of discordant results between 4 WHO approved RT-PCR protocols to detect the SARS-CoV2 genome: BERLIN, HKU, DAAN gene and IPP, through testing 260 real clinical specimens. The DAAN gene® commercial test used the same CDC China protocol approved by the WHO. In vitro analysis showed discordant results in 29.2% of cases (76 out of 260). The most discordant protocol is DAAN Gene® due to false positive late signals with N target. Discordant results between the two protocol’s targets are more frequent when viral load are low (high Ct values). Our results demonstrated that the multiplexing has worsened the sensitivity of the IP4 target. We provide concise recommendations for the choice of the targets, the interpretation of the results and the alarm signals which makes suspect a gene mutation.

Globally, molecular testing by PCR has revolutionized the diagnosis of infectious diseases. In the context of the COVID-19 pandemic, high performing tests has allowed it to identify infected people and to decide of their discharge. Different targets were proposed and validated, classically; the preferred targets of pathogens include the conserved specific genes as the nonstructural genes like RdRp or genes that are expressed abundantly such as the structural S and N genes. In our study, the protocols were approved since March 2020 by the WHO. Many authors studied these protocols to focus on sensibility and specificity by in vitro or in silico studies [10–12]. Our study explored in vitro clinical specimen. Importantly, our study used a large number of clinical specimens from patients with confirmed COVID-19, oppositely, the majority of published studies explore cell culture supernatant or RNA transcripts, or sometimes a very small number of real samples collected in a precise time [13].

In our study we found that 29.2% of tested samples gave discordant results (76 out of 260), occurring more often with high Ct values and probably due to false positive amplifications. Sule et al*.* suggested that the Ct values over than 28 are probably related to non-specifically precipitated sequences due to an inactivation of the Taq polymerase and proposed that Ct values > 33.33 or 35, or ≥ 39.2 or 40 could be considered as negative [14]. In our study, Ct value less than 40 is considered positive, according to the respective protocol authors.

Among all discordant cases, false negative results represent the most disturbing cases, for our study we obtained 21 samples positive by 3 protocols but one (Group D1 Table 2). This is problematic because not only it may underestimate the COVID-19 incidence but, perhaps more acutely, will lead to infectious individuals remaining as a source of infection in the community and undermine the effectiveness of infection control measures. Clinicians should not hesitate to re-sample the high suspected patients when laboratories return negative result.

The N gene target was the most problematic. From the one hand, this is due to the false negative results caused by mismatches with the primers and probes [15]. Thanks to many in vitro and in silico analysis, researchers around the world are tracing the ongoing evolution of the N gene and demonstrated that it was particularly prone to mutations, more than all other targets. These findings affected the HKU, CDC China and Japan NIID N targets [11, 15–17]. Wang et al. concluded in 14 september 2020 that the N gene is the most non conservative gene giving non uniform performances between different primers and probes, thus, the N gene may not be an optimal choice [11]. This has become more preoccupant after the emergence of the Alpha VOC that caused a N gene dropout and N gene Ct value shift comparing to the wild strain [18]. For the other hand, N gene target was widely criticized to give persistent late signals for convalescent patients, which make confusion between false positivity or low amount of virus, related to convalescent patients [19, 20]. More importantly, this caused a dilemma regarding the discharge of isolation policies making the WHO canceling the need to 2 negative RT-PCR results [21]. Moreover, soon after the publication of the United States of America CDC protocol, Lee et al. declared that the N gene target should be canceled and disused because of false positive reactivity of N3 [22] This was found in our study for the DAAN gene® N target with late Ct values, which underlines that late positive signal for N gene alone is more likely to be false positive and needs to be interpreted with caution. In our series, the HKU N target didn't show so many false positive results; this would be related to the primers and probes design as they do not target the same region in the N gene.

Since its publication in January 2020, the BERLIN protocol was widely used worldwide [5]. It was reported that the E gene was the most sensitive and the most conservative target [11, 23] apart from some mutations affecting its sensitivity leading to false negative results by a commercial test using this protocol [10].

BERLIN protocol was criticized for the low sensitivity of the RdRp target which was proposed to confirm all BERLIN E gene positive results [24, 25]. Our results demonstrated that, in fact, 15 positive samples by BERLIN E target could not be confirmed by BERLIN RdRp target, in addition, BERLIN E gene gaves significantly lower Ct values than BERLIN RdRp leading to negative results for BERLIN RdRp when BERLIN E gaves late Ct values.

It is obvious that multiple RT-PCR reactions require more reagents, controls, thermo cyclers and labor, not adapted to a pandemic context. Multiplexing offers the possibility of two or more target detection by just one reaction, which have become attractive thanks to reducing significantly reagent consumption and time. Many authors suggested and demonstrated many successful multiplexing assays, without substantially reduce the test performances [26–28]. However, in our study we demonstrated for the first time that multiplexing has drastically decreased the sensitivity of the IP4 target for particular samples. On 18 March 2021, the Centre National de Référence Virus des Infections Respiratoires (CNR), the authors of the IPP protocol, has reported a loss in the sensitivity of the IP4 target due to a mutation in the Orf1ab(C14050T), associated to the Alpha VOC [29]. Our study samples were not sequenced, but in the routine surveillance of our lab, we noted that many samples with IP2+/IP4− belong to Alpha VOC, mainly detected by partial sequencing in the S gene [30] and variant-specific RT-PCR tests SNPsig®real-time PCR SARS-CoV-2 mutation detection/allelic discrimination kit (Primerdesign Ltd; UK). Indeed, the samples with low Ct values with IPP IP2 target and negative amplification with the IPP IP4 target were collected between Week-7 and Week-16 of the year 2021; which corresponds to the high transmission period of the Alpha VOC in Tunisia (Chouikha et al. [31]. The CNR IPP has published later in the 6 April 2021 a new RT-PCR Mix to overcome the false negative results. We demonstrated by experience that this abnormality was resolved by using the same targets in singleplex. This may be explained by a competition in the reaction which makes IP2 a preferred target and inhibits IP4 annealing. For this reason any multiplexing should be explored versus singleplex. Regarding the other targets, it was already reported that the Alpha VOC emergence has affected the sensitivity of some tests that amplify in the Spike gene, due to the deletion 69–70 [32]. A large in silico study based on the theoretical production of RT-PCR signals by the SCREENED software, has evaluated the impact of the Alpha VOC on the RT-PCR protocols used in our study; the authors detected only one mutation (C12778T) in the Alpha VOC within the IPP IP2 target amplicon that has no impact on its sensitivity, as it is not located in the primers and probes annealing sites [33].

Our study argued the need of using at least two independent virus key regions to avoid the false positive and negative results. The N gene is better to be avoided or should be interpreted with much caution. Otherwise, N late signals should be confirmed by testing another non structural region. The E target is the best one in terms of specificity; it presents the best agreement with non structural targets like IP2 and HKU Orf 1b with significant lower Ct values which may substitute the BERLIN RdRp to confirm positive E samples. Biologists should be aware about some alarm signals that should indicate close monitoring and investigating technical or molecular causes i.e. when using IPP or HKU protocol, any divergence between targets of more than 2 Ct values, when one target gives high positive signal while the other is totally negative and when a gene dropout is obtained for gene multiplex assays. Here, re-testing in singleplex should be performed as well as indicating sequencing. As the introduction of variants has worried the researchers about the reliability of RT-PCR protocols, which were established at the beginning of 2020 before the emergence of variants mutations, we recommend that all laboratories perform regular in silico analysis in order to assess the test performance. This could be limited for laboratories using commercial tests that do not specify exactly the primers and probes used.

## Conclusion

In conclusion, the data presented in this study show the importance of regularly assessing the used RT-PCR protocols especially in the context of new emerging variants. Our findings emphasize that although WHO approved, RT-PCR protocols should be evaluated by each laboratory. Molecular testing by RT-PCR is a double trapped weapon that any mis-interpretation may lead to false positive or negative results, impacting the COVID-19 surveillance.

## Data Availability

Not applicable.
